# Advances in targeting and heterologous expression of genes involved in the synthesis of fungal secondary metabolites

**DOI:** 10.1039/c9ra06908a

**Published:** 2019-10-30

**Authors:** Yun-Ming Qiao, Rui-Lin Yu, Ping Zhu

**Affiliations:** State Key Laboratory of Bioactive Substance and Function of Natural Medicines, NHC Key Laboratory of Biosynthesis of Natural Products, CAMS Key Laboratory of Enzyme and Biocatalysis of Natural Drugs, Institute of Materia Medica, Chinese Academy of Medical Sciences, Peking Union Medical College Beijing 100050 China zhuping@imm.ac.cn +86-10-63017757 +86-10-63165197

## Abstract

The revolutionary discovery of penicillin only marks the start of our exploration for valuable fungal natural products. Advanced genome sequencing technologies have translated the fungal genome into a huge reservoir of “recipes” – biosynthetic gene clusters (BGCs) – for biosynthesis. Studying complex fungal genetics demands specific gene manipulation strategies. This review summarizes the current progress in efficient gene targeting in fungal cells and heterologous expression systems for expressing fungal BGCs of fungal secondary metabolites.

## Abstract 2

Gene targeting involves integration of foreign DNA into the fungal genome by several strategies including *Agrobacterium tumefaciens*-mediated transformation (ATMT).

## Graphical abstract

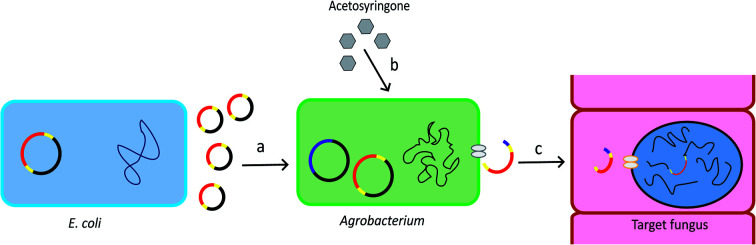

## 1 Introduction

The fungal metabolome is an important source of natural products, including drugs (*e.g.* ergometrine), antibiotics (*e.g.* penicillin), agricultural and industrial products (*e.g.* strobilurin), and food additives (*e.g.* azaphilones).^1^ Almost all valuable fungal products are secondary metabolites (SMs) and are defined as organic molecules synthesized for purposes other than growth, development, or reproduction.^2^

A lot of genes that encode for enzymes involved in the synthesis of SMs are arranged in biosynthetic gene clusters (BGCs) in the fungal genome, such as the ergot alkaloid BGC.^3,4^ Today, over 1000 fungal genomes are available.^5,6^ Conservative estimates suggest that the fungal genomes contain over several million BGCs.^7^ Moreover, since the number of BGCs far exceeds the number of known SMs, fungal genomes have immense potential for discovery of novel natural products.^7^

Understanding of each gene within a BGC would allow for total biosynthesis of the theoretical SM, engineering of the biosynthetic pathway, and a more reliant source of the SM for chemical derivation or other research. However, the complex biology of filamentous fungi is delaying discovery and biosynthesis of fungal SMs. Fungal BGCs can be tightly regulated or completely silent in their native species, which explains the disparity between the number of predicted BGCs and known SMs.^1^ For example, the plant endophytic fungus *Dothideomycete* sp. CRI7 exhibited the ability to produce a novel tricyclic polyketide and its biosynthetic precursor azaphilone derivatives.^8^ While simple changes such as culture media or an addition of halogen salts enabled this endophytic fungus even to produce new related compounds,^9,10^ suggesting that there are many silent genes in fungi.

Compared with prokaryotic systems, filamentous fungal systems are much more complex, frequently facing problems such as inefficient transformation and uncharted, complicated genetic background. Uncovering the functions of genes involved in a fungal BGC often includes the following two aspects:

(1) Gene targeting (deletion, disruption, or integration of a certain gene) in BGCs within the native fungus.

(2) Heterologous expression that circumvents the complex fungal system. With the development of biosynthetic pathway engineering, heterologous expression in other microbial hosts is also more economic and sustainable than culturing fungi.

Previous reviews have summarized many techniques for fungal genetic studies, but they did not pay much attention to single gene or single BGC manipulation techniques.^11,12^ This review focused on recent progress in methods specifically for individual fungal gene or fungal BGC targeting and heterologous expression, as exemplified by ergot alkaloid biosynthesis.

## 2 Gene targeting

Gene targeting refers to the replacement or inactivation of the gene of interest. It is very useful for studying biosynthetic pathways. Gene targeting involves the integration of foreign DNA by the cellular DNA double strand repair mechanisms: homologous recombination (HR) or non-homologous end joining (NHEJ). In nature, DNA double strand break (DSB) is the most severe type of DNA damage.^13^ The HR achieves targeted integration, but most filamentous fungi favour the NHEJ repair that joins together two free DNA ends regardless of the homology. Therefore, in gene targeting experiment the NHEJ almost always results in ectopic integration or unsuccessful targeting.^13^ Thus, gene targeting in fungi through direct molecular transformation is often inefficient with frequency of HR as low as <1%.^14–16^ HR frequency naturally varies between species and due to modifications in experimental factors. Data from gene targeting studies are summarized in Table 1.

**Table 1:** Examples of gene targeting outcomes^a^

Method	Host fungi	Target locus	Before	After	Notes	
NHEJ impair – Ku disruption	*Claviceps purpurea*	*lpsA1*	1%	64%	Increased sensitivity to certain antibiotics	21
	*Penicillium chrysogenum*	*hdfA*	1%	47%	Ku mutant showed reduced fitness when co-cultivated with the wild-type	17
		*hdfB*		56%		
	*Penicillium decumbens*	*creA*	33%	100%		23
		*xlnR*	91%	100%		
	*Penicillium marneffei*	*10 loci*	0.42%	70%	Genome instability at *pkuA* locus; various phenotype differences	20
	*Trichoderma virens*	*lcc1*	15%	88%		24
NHEJ impair – ligD disruption	*Aspergillus kawachii*	*argB*	0%	6%, 65%, 90%	Homology arm size = 500, 1000, 1500 bp	26
	*Penicillium chrysogenum*	*mre11*	0%	25%	Numbers are estimates from graph	16
		*niaD*	5%	70%		
		*rad50*	5%	40%		
	*Penicillium oxalicum*	*PoargB*	19%	97%	Homology arm size = 500 bp	25
		*PoagaA*	73%	90%		
		*Podpp4*	0%	27%		
ATMT	*Aspergillus oryzae*	*pyrG*	—	0.001–0.01%		33
	*Claviceps paspali*	*idtCBGF*	0%	25.00%		66
	*Ustilaginoidea virens*	*Uvhog1*	0%	0.16%		30
	*Aspergillus carbonarius*	*ayg1*	3%	18.00%*	*: Best result obtained using *Agrobacterium* AGL-1 strain	34
CRISPR-Cas9	*Aspergillus carbonarius*	*ayg1*	3%	1%		34
	*Neurospora crassa*	*clr-2*	—	21.43%		46
	*Penicillium chrysogenum*	*pks17*	—	60.00%	Homology arm size = 1000 bp	47
	*Pyricularia oryzae*	*SDH*	0%	*9.8–80.5%	*Depending on targeting sites and choice of promoter	48
	*Trichoderma reesei*	*lae1*	—	93%	Homology arm size = 200 bp	41
	*Ustilago maydis*	*bE1*, *bW2*	0%	30–100%		49
		*bW2*	0%	8–100%		
NHEJ impair + ATMT	*Metarhizium robertsii*	*Cag8*	6.5%	93.4%		64
	*Penicillium digitatum*	*PdchsII*	0.60%	11.40%	Reduced growth at >28 °C; reduced conidia production at >26 °C	22
		*PdchsV*	0%	6.60%		
NHEJ impair + CRISPR-Cas9	*Aspergillus aculeatus*	*albA*	0%	*	*: “most of the transformants”	67
	*Aspergillus nidulans*	*yA*	0%	90%		
ATMT + CRISPR-Cas9	*Ganoderma lucidum*	*ura3*	—	67–100%		45
	*Ganoderma lingzhi*		—	88.89%		
	*Sporisorium scitamineum*	*Mfa2 (knock-out)*	—	27.1–39.1%	Cas9 and targeting cassette introduced at the same time	65
		Mfa2 (knock-in)	—	74.50%	Cas9 present before introduction of targeting cassette	

a “—” indicates data omitted in the paper. “Before”: frequency of precise gene targeting in wild-type (NHEJ impair) or protoplast-mediated transformation (ATMT). “After”: frequency of precise gene targeting after NHEJ impair, ATMT, or CRISPR-Cas technology.

### 2.1 Impairing the non-homologous end joining (NHEJ) pathway

The gene targeting efficiency in most filamentous fungi is generally extremely low due to predominant random integration caused by the non-homologous end-joining (NHEJ) pathway. Therefore, a common practice is to inactivate the NHEJ pathway by deleting the fungal homologous of the human *ku70* and *ku80* genes that encode for the Ku protein complex.^17^ The NHEJ mechanism involves the binding of the dimeric Ku to the free DNA ends to form a ring encircling DNA. Then, the Ku protein complex recruits and mediates other proteins or catalytic subunits for DNA repair, including the DNA-dependent polyketide catalytic subunits (DNA-PKcs) and the DNA ligase IV-XRCC4 complex.^18,19^ The drawbacks of impairing the NHEJ can include reduction in the total transformation frequency (total transformation = NHEJ + HR)^17^ and generation of various side effects on cell phenotype.^20,21^ Also, impairing the NHEJ is very difficult to achieve in fungi with low transformation efficiency.

Impairing the NHEJ *via ku* disruption is generally effective in the genus *Penicillium*.^16,20,22,23^*Penicillium chrysogenum* is a filamentous fungus used for industrial production of β-lactam antibiotics. Deletion of the *ku70* and *ku80* homologues in *P. chrysogenum* resulted in mutants with improved HR frequency (from 1% to 40–50%). The total transformation frequency was lower because the cells could not gain chlorate resistance through NHEJ. The *ku70*-knockout approach also significantly improved the frequency HR in *Claviceps purpurea*^21^ and *Trichoderma virens*.^24^

Impairing the NHEJ mechanism through *ku* knockout might have unintended physiological alterations on fungi. Fortunately, most studies reported moderate to no differences. In the study on *P. chrysogenum*, the Δ*hdfA* mutant (*ku70* knockout) and the wild-type strain had similar biomass yield, CO_2_ production, O_2_ consumption, penicillin G production, and similar response to phleomycin, an antibiotic that induces DNA double-strand breaks. However, the Δ*hdfA* mutant showed reduced fitness when it was co-cultivated with the wild-type strain.^17^ The Δ*ku70* mutant of *C. purpurea* showed the wild-type phenotype in growth rate, germination rate, pathogenicity and alkaloid production, but the mutant had moderately increased sensitivity to several antibiotics, including phleomycin, hygromycin and nourseothricin. Increased sensitivity toward phleomycin was expected because phleomycin induces DNA double strand break, but the reason for growth repression due to hygromycin and nourseothricin was not clear.^21^ Between the *T. virens* Δ*tksu70* mutants and the wild-type control, there was no statistically significant difference in phenotype in terms of growth rate, conidial germination, antagonistic properties, growth under osmotic stress, or growth under DNA damaging agents.^24^

Since DNA ligase IV (Lig4) is also important to the NHEJ mechanism, disruption or deletion of *lig4* is another way of generating NHEJ-impaired fungal cells. Disruption of *lig4* has successfully improved the frequency of HR in *Penicillium oxalicum*,^25^*P. chrysogenum*,^16^ and *Aspergillus kawachii*.^26^ For some species such as *Neurospora crassa*, disruption of *lig4* is more effective than disruption of *ku* in improving HR frequency because their NHEJ pathways involve *Lig4* but not *Ku*.^27^

### 2.2 *Agrobacterium tumefaciens*-mediated transformation (ATMT)

**Fig. 1:**
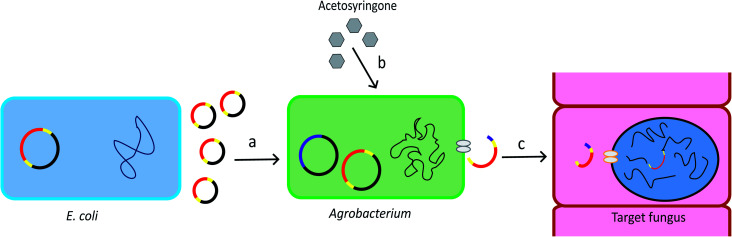
Procedure of ATMT. (a) The T-DNA plasmid can be produced by recombinant *E. coli.* The plasmid is then transformed into *Agrobacterium* cells. Yellow: T-DNA borders. Red: gene of interest. (b) Acetosyringone induces expression of the *vir* genes which are located in the bacterial chromosome and in the helper plasmid. Pink: *vir* genes. (c) The *vir* genes direct the copy, gene transfer and incorporation of the T-DNA into the target cell.^28^

*Agrobacterium tumefaciens* is a Gram-negative soil bacterium. It introduces tumour to plants by transferring part of its tumour-inducing (Ti) plasmid, the Transfer-DNA (T-DNA), into the plant genome. The development of the T-DNA binary vector system allows for convenient introduction of the gene of interest into the bacterium T-DNA.^28^ The *Agrobacterium* genus contains strains that can transform a wide range of host cells, including plants, fungi, and mammalian cells.^29^ The schematic overview of ATMT system is provided in Fig. 1.

As a gene targeting strategy, *Ag. tumefaciens*-mediated transformation (ATMT) has numerous advantages. ATMT can be used for purposes ranging from individual gene deletion or integration^30^ to generation of hundreds of mutants.^31,32^ It is more likely to integrate a single copy of gene than restriction enzyme mediated transformation (REMI), allowing precise genetic manipulations sequentially in the same locus.^28^ In addition, ATMT is applicable to many fungal species, removing the restriction on using model species for molecular biology experiments.^28^ Thus, ATMT is widely used in the production of transgenic crops and in the plant and fungal genetic studies.^29^ A disadvantage of ATMT is that it is time-consuming because it requires optimization of transformation conditions, such as optimization of ratio of host cells to the bacteria, co-cultivation temperature, and co-cultivation time.^12^

ATMT has successfully targeted genes in a variety of fungi. *Ustilaginoidea virens* causes rice false smut and harms the nervous system of animals. *U. virens* is closely related to *C. purpurea* – both known for low HR frequency. ATMT improved the frequency of targeted integration from 0% in PEG-mediated protoplast transformation to 0.1%. The study was the first successful attempt of targeted gene deletion in *U. virens*.^30^*Aspergillus oryzae* is a filamentous fungus with important role in the food industry and recombinant protein production. Because *A. oryzae* is resistant to many antifungal antibiotics, transformants are typically screened by auxotrophic characteristics. ATMT was used to produce useful uridine/uracil auxotrophic *A. oryzae* mutants. Consequently, 11-1060 *pyrG*-deleted, uridine/uracil auxotrophic transformants were generated from 10^6^ spores (0.001–0.1%).^33^

*Aspergillus carbonarius* is a fungus capable of producing many industrially relevant biochemical products. Three methods were used to disrupt *ayg1* in *A. carbonarius*: (1) ATMT, (2) protoplast-mediated transformation (PMT) using bipartite fragments or (3) CRISPR-Cas9. The efficacy of each method was evaluated by the frequency of precise targeting over total generated transformants (HR/(HR + NHEJ)). Bipartite gene targeting fragments were synthesized *via* PCR; they contained incomplete transformation marker that would only become functional if it was integrated into the right location in the fungal genome. PMT of bipartite fragments or CRISPR-Cas9 yielded higher frequency of transformation but lower frequency of HR, compared with ATMT. While ATMT using the *Ag. tumefaciens* AGL-1 strain yielded the highest (18%) frequency of HR. Contrarily, the *Ag. tumefaciens* LBA4404 strain failed to disrupt the target gene (0%). The study showed that ATMT efficiency depends heavily on the choice of *Agrobacterium* strain.^34^ However, another study aimed to generate a mutant library using ATMT discovered that ATMT efficiency was not affected by the choice of *Ag. tumefaciens* strain.^32^ This could imply that the choice of *Ag. tumefaciens* strain likely affects gene targeting rather than genome studies without a gene target.

### 2.3 CRISPR-Cas9 transformation

The CRISPR (clustered regularly interspaced palindromic repeats) – Cas9 (CIRSPR associated protein9) system is a gene editing tool derived from the bacterial cell defence system. In 2013, it was first shown to precisely edit mammalian cell genome.^35^ Almost immediately, CRISPR-Cas9 became a common practice in genome editing for its easy programmability, low cost, and wide applicability.

The CRISPR-Cas9 system contains two crucial components: the Cas9 nuclease and sgRNA (single-guide RNA, a synthetic fusion of small CRISPR RNA (crRNA) and trans-activating crRNA (tracrRNA)). In this system, an about 20 bp fragment of the partial sgRNA recognizes the target genomic DNA *via* Watson–Crick base pairing. Cas9 can then bind to target DNA *via* a 3 bp proto-spacer adjacent motif (PAM) sequence in the target DNA downstream of the region.^36^ The Cas9 enzyme induces double strand breaks (DSB) in the genomic DNA which can be repaired by nonhomologous end joining (NHEJ) or homologous recombination (HR); the latter requires a DNA repair template (donor DNA).^37^

The CRISPR-Cas9 technique has been used extensively as a gene manipulation tool in many types of cells.^38–40^ However, due to the complexity of filamentous fungal cells, such as multinuclear structure, cell differentiation and thick chitin cell wall structure, as well as the difficulty of genetic operation, the CRISPR/Cas9 system has not been widely applied in filamentous fungi until the successful establishment of the genome-editing approach in *Trichoderma reesei*. In 2015, a CRISPR/Cas9 system capable of gene editing for specific targets in *T. reesei* genome was successfully constructed.^41^ DNA sequence for expressing Cas9 nuclease was optimized and transformed into the fungus using ATMT. Due to a lack of suitable promoter, sgRNA was transcribed *in vitro* and introduced directly to cells expressing Cas9. For targeted disruption of *lae1*, HR frequency reached 93% using homology arm size as small as 200 bp. Frequency of double recombination reached 45% after adjustment in the two sgRNA and respective donor DNA. This study showed that CRISPR-Cas9 can be used for genome engineering in fungi.^41^ In the following years, CRISPR-Cas9 systems have been successfully constructed in *Aspergillus fumigatus*,^42^*A. oryzae*,^43^*Coprinopsis cinerea*,^44^*Ganoderma lingzhi*,^45^*N. crassa*,^46^*P. chrysogenum*,^47^*Pyricularia oryzae*^48^ and *Ustilago maydis*,^49^ apart from the monocellular *Saccharomyces pyogenes*,^50^*Saccharomyces cerevisiae*,^51^ and *Candida albicans*.^52^

However, some disadvantages of CRISPR/Cas9 gene editing system should be emphasized: first, appropriate promoters are required for the expression of the sgRNA and Cas9 protein. The poly III promoter U6 was usually used to activate the expression of sgRNA. When the U6 promoter is not available, other promoters were also used for expressing gRNA in various fungi.^42,47–49^ However, available promoters are ill defined in some filamentous fungi such as *T. reesei*,^41^*P. chrysogenum*,^47^*A. fumigatus*,^42^*Aspergillus niger*^53,54^ and *Beauveria bassiana*,^55^ for which the sgRNAs were synthesized *in vitro*.^41^ Codon optimized Cas9 has been applied in many filamentous fungi and can enhance the genome editing efficiency. Moreover, the inducible or constitutive promoters can activate the Cas9 expression. Second, lack of selection markers limits genetic manipulation in wild-type filamentous fungi.^36^ Common markers for screening positive mutants include hygromycin resistance gene (*hph*), pyridine thiamine resistance gene (*ptrA*), phleomycin resistance gene (*zeo*), and glufosinate-ammonium resistance gene (*bar*). However, the limited number of selective markers cannot satisfy systematic genetic modification of the same strain in multiple directions. Currently, some site-specific recombination systems have been successfully used to recover and reuse selective markers, such as β rec/*six* systems from prokaryotes,^56,57^ Cre/*loxP* system from phage P1 ^58–60^ and Flp/*FRT* system from *S. cerevisiae*.^61,62^ The subsequent frequency of cassette eviction usually reached 100% and not affected the frequency of gene replacement by homologous recombination.^57^

The third disadvantage of CRISPR-Cas9 system is that it might trigger mutation in off-target sites when either Cas9 binds to PAM-like sequence, or sgRNA binds to sites with high homology to target DNA.^36^ Algorithms such as Cas-OFFinder have been developed to predict off-target cleavage sites, but it is impossible to identify every off-target site with such algorithms.^63^ Therefore, alternative strategies are needed to improve the specificity of Cas9, such as modifying the enzyme or editing the sgRNA.^36^

### 2.4 Using suitable genetic manipulation method or a combination of methods

The gene *ayg1* is involved in the melanin synthesis pathway in *A. carbonarius*. Loss of *ayg1* leads to change in spore colour. Gene knock-out of *ayg1* in *A. carbonarius* was performed using PEG/CaCl_2_ transformation method, ATMT method and CRISPR-Cas9 gene targeting method in order to compare the efficiency of each method.^34^ The targeting efficiency of ATMT method was as high as 27%, while that of the CRISPR-Cas9 method was less than 1%. Therefore, screening of the most suitable genetic manipulation method is necessary for each specific filamentous fungus species.

Using ATMT to generate NHEJ-impaired mutants is an effective method for gene targeting in fungi, such as *Metarhizium robertsii*, whose transformation efficiency is generally low because of the NHEJ mechanism. Gene targeting plasmid was generated by the 2 day One Step Construction of *Agrobacterium* Recombination (OSCAR) method. *M. robertsii* cells were first transformed by ATMT to yield NHEJ-impaired mutant (Δ*MrKu70*). The Δ*MrKu70* mutant was then transformed by the gene targeting plasmid for *Cage8* disruption. The frequency of HR was improved from 6.5% in the wild-type strain to 93.4% in Δ*MrKu70*. The gene targeting plasmid generated by the OSCAR can also be applied to other fungi with modifications such as the choice of resistance gene.^64^

Another combined method is to use ATMT to transform CRISPR-Cas9 system into fungi. This combination enabled successful site-specific knock-out and complementation knock-in in *Sporisorium scitamineum*. *Mfa2* knock-out mutants were generated with 21.7–39.1% frequency of targeted gene disruption, depending on different target sites in *Mfa2*. To achieve knock-in without triggering the CRISPR-Cas9 system, *Mfa2* knock-out mutants were transformed by the modified *Mfa2* gene harbouring synonymous mutations. This generated *Mfa2* knock-in mutants with 74.5% frequency of targeted integration. The presence of Cas9 prior to introduction of the targeting cassette was the reason for increased frequency of integration in the second transformation.^65^

## 3 Heterologous expression systems

Heterologous expression (HE) of a gene of interest followed by catalytic reactions is an effective way of determining gene function. HE of fungal BGCs facilitates development of industrial production for valuable fungal secondary metabolites. The general procedure begins with amplification of gene of interest and vector construction, followed by vector design, transformation, culture and expression, optional protein purification depending on how the reaction for verification of enzyme function is to be conducted. This section reviewed the research progress in heterologous expression of the fungal genes or BGCs involved in fungal secondary metabolic pathways. A summary of research outcomes of different fungi genes in different heterologous expression systems is provided in Table 2.

**Table 2:** Heterologous expression outcomes^a^

Host organism	Gene source	Gene name	Protein	Biosynthesis product	
*Escherichia coli*	*Aspergillus flavus*	*BVMOAFL706, 570*, *334*, *778*	Type I Baeyer–Villiger monooxygenase	Lactones	93
	*Penicillium roqueforti FM164*	*fgaOx3Pr3*	Chanoclavine-I aldehyde reductase	Festuclavine (EAS pathway)	73
	*Penicillium roqueforti DSM1079*	*fgaDHPr*	Festuclavine dehydrogenase		
	*Penicillium camemberti DSM1233*	*fgaDHPca*			
	*Hebeloma cylindrosporum*	*hcLAAO4*	l-Amino acid oxidase	α-Keto acids	94
	*Aspergillus oryzae*	*agl1*	α-1,6-Glucosidase (isomaltase)	Glucose	95
	*Aspergillus niger*	*agdC*			
	*Fusarium oxysporum*	*foagl1*			
	*Penicillium herquei*	*hqlA*	Non-ribosomal peptide synthetase	Pyrazine	96
*Saccharomyces cerevisiae*	*Aspergillus japonicus*	*easC_Aj*	Catalase	Chanoclavine-I (EAS pathway)	77
		*easE_Aj*	FAD-dependent oxidoreductase		
	*Aspergillus japonicus*	*easD*	Oxidase	Cycloclavine (EAS pathway)	78
		*easA and easG*	Reductase		
		*easH*	Dioxygenase		
	Diverse ascomycete and basidiomycete fungal species	—	22 BGCs with polyketide synthase or UbiA-type cyclase at its core	—	68
	*Xylaria grammica*	*frlA*	P450 monooxygenase	Statin	97
		*frlB*, *frlF*	Polyketide synthase		
		*frlC*	Enoyl reductase		
		*frlD*	Transesterase		
		*frlG*	Thioesterase		
		*frlH*	HMG-CoA reductase		
	*Aspergillus nidulans*	*npgA*	Phosphopanetheinyltransferase		
*Aspergillusnidulans*	*Aspergillus fumigatus*	*dmaW*	Dimethylallyltryptophan synthase	Chanoclavine-I (EAS pathway)	80
		*easF*	Methyltransferase		
		*easE*	Oxidoreductase		
		*easC*	Catalase		
	*Aspergillus terreus*	—	9 nonreducing polyketide synthases	—	81
		*AteafoA*	Regulator	Asperfuranone	
		*AteafoB*	Efflux pump		
		*AteafoC*	Esterase/lipase		
		*AteafoD*	Salicylate monooxygenase		
		*AteafoE*	Nonreducing polyketide synthase		
		*AteafoF*	FAD-dependent oxidase		
		*AteafoG*	Highly reducing polyketide synthase		
*Baccilussubtilis*	*Fusarium oxysporum*	*eysn*	Non-ribosomal peptide synthetase	Enniatin	86
*Aspergillus fumugatus*	*Epichloë* sp. Lp1.	*easA*	Isomerase	Agroclavine (EAS pathway)	87
		*easH*	Dioxygenase	Lysergic acid (EAS pathway)	
		*cloA*	Clavine oxidase (P450 monooxygenase)		
*Aspergillus oryzae*	*Monascus pilosus*	*mokA*, *B*	Polyketide synthase	Monacolin K	88
		*mokC*	P450 monooxygenase		
		*mokD*	Oxidoreductase		
		*mokE*	Dehydrogenase		
		*mokF*	Transesterase		
		*mokG*	HMG-CoA reductase		
		*mokH*	Probable transcription factor		
		*mokI*	Efflux pump		
	*Aspergillus nidulans*	*tdiA*	Non-ribosomal peptide synthase	Terrequinone Q	
		*tdiB*	Indoleprenyltransferase		
		*tdiC*	Oxidoreductase		
		*tdiD*	PLP-dependent transaminase		
		*tdiE*	Unknown protein		
*Aspergillus niger*	*Trichoderma virens*	*cbhI*	Cellobiohydrolase	Cellobiose	90
	*Aspergillus terreus*	*terA*	Non-reducing peptide synthase	Orsellinic acid, 6,7-dihydroxymellein and 4-hydroxy-6-methylpyrone (terrein pathway)	98
		*PamyB:terR*	Transcriptional regulator		
*Neurospora crassa*	*Aspergillus terreus*	*CAD1*	*cis*-Aconitic acid decarboxylase	Itaconic acid	91
*E. coli-based cell-free system*	*Brevibaccilus brevis*	*grsA* and *grsB1*	Non-ribosomal peptide synthetase	Gramicidin S	92

a “—” indicates too much text to fit in the table.

### 3.1 Host organism: *Escherichia coli*

HE in *E. coli* is an effective approach for studying many fungal proteins. Using *E. coli* as the host organism has many advantages: (1) *E. coli* has fast growth (2) high cell density can be reached easily (3) cultivation medium is cheap and simple (4) transformation procedure is fast and easy. Large-scale protein expression trials have shown that 50% bacterial protein and 15% non-bacterial proteins can be expressed in *E. coli* in a soluble form.^69^

Despite being the most popular prokaryotic HE system, *E. coli* has many limitations that include inability to perform post-translational modifications, to fold eukaryotic proteins correctly which may lead to formation of inclusion bodies of insoluble proteins, or to recognize promoters and dismiss terminators and introns in filamentous fungal genes.^70^ Codon bias, the inefficiency of tRNAs in *E. coli* to recognize certain codons in the foreign gene, could also reduce protein expression. The need to replace fungal promoters with host promoters, removal of terminators and introns, and codon optimization are laborious tasks which make *E. coli* a less suitable HE host, especially for expression of large fungal gene clusters.^69^

Many parameters need to be considered for successful HE in *E. coli*, such as the choice of host strain and specificities in vector construction.^69^ At the transcriptional level, gene copy number, promoter strength, and induction environment should be carefully selected to ensure slow and controlled expression that promotes correct protein folding. At the translational level, HE is affected by mRNA ribosomal binding site (RBS) secondary structure, the 5′ UTR sequence of mRNA, availability of tRNA and free amino acids, and the presence of regulator genes that affect translation rate. The use of fusion tags has benefits including easier purification, improved protein solubility, and increased mRNA stability. To further improve expression, one can consider cofactor regeneration, the use of chaperones to prevent protein mis-folding, to improve protein export, and to overcome feedback inhibition from product formation.^71^

The biosynthetic pathways of clavine-type alkaloids or d-lysergic acid from the branch point chanoclavine-I aldehyde in different species have been described in detail elsewhere^72^*. E. coli* HE system has been used for characterizing genes from the ergot alkaloid biosynthetic gene cluster of *Penicillium roqueforti* FM164. The *fgaOx3*_*Pr3*_ gene was identified by computer-assisted analysis on *P. roqueforti* genome using sequences from the ergot alkaloid biosynthetic gene cluster of *A. fumigatus*. The size and conformation of the purified recombinant protein was determined. Enzyme activity assays confirmed that FgaOx3_Pr3_ is a chanoclavine-I aldehyde reductase that converts chanoclavine-I aldehyde to festuclavine in the presence of festuclavine synthase FgaFS or the agroclavine synthase EasG. FgaOx3_Pr3_ also improved the conversion of the previous step in the ergot alkaloid synthetic (EAS) pathway by overcoming product inhibition of chanoclavine-I dehydrogenases (FgaDH_Pr_ and FgaDH_Pca_).^73^

### 3.2 Host organism: *Saccharomyces cerevisiae*

*S. cerevisiae*, also known as the Baker's yeast, is a unicellular eukaryotic organism that is routinely used as host for HE of fungal genes. *S. cerevisiae* has many advantages including fast growth, cheap and simple culture media, well-studied and accessible genetic tools, ease of gene manipulation, and eukaryotic post-translational modifications.^74^

Conditions for HE in *S. cerevisiae* can be optimized depending on the yeast strain and the recombinant protein. Examples of these conditions include: culture medium pH, cell density during fermentation, and oxygen level. Yield of HE can also be improved by genetic manipulation in native yeast genes which are involved in transcription and translation, such as overexpression of protein disulfide isomerases (PDIs) or other chaperones, and metabolic engineering.^75,76^ Harvey *et al.* demonstrated that 22 out of 41 selected fungal BGCs have been expressed in an optimized *S. cerevisiae* HE platform (HEx). The platform consists of a method for refactoring fungal BGC, an engineered yeast strain, and a yeast HR strategy that allows assembly of up to 14 fungal genes.^68^

**Fig. 2:**
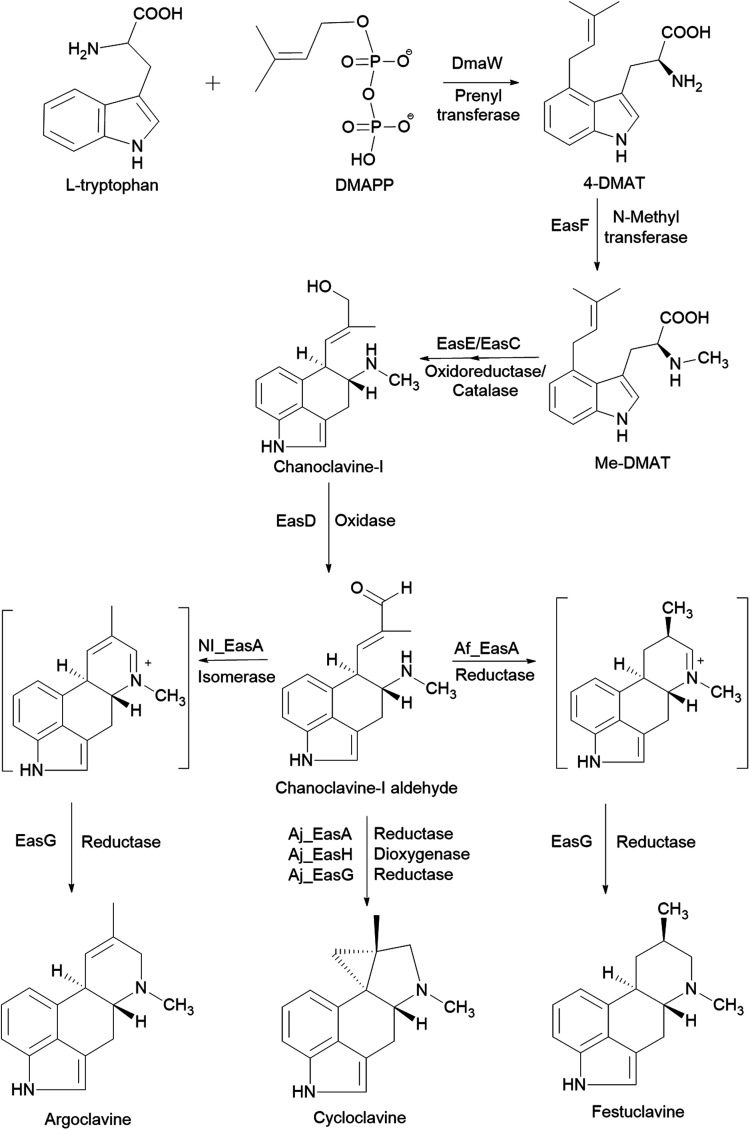
The recombinant biosynthetic pathway of festuclavine, agroclavine, and cycloclavine from l-tryptophan and DMAPP in *Saccharomyces cerevisiae*.^78^

*S. cerevisiae* is often used for studies on the fungal pathway of ergot alkaloid synthesis (EAS). The formation of chanoclavine-I from l-tryptophan and dimethylallyl pyrophosphate (DMAPP) under DmaW, EasF, EasC and EasE catalysis was reconstituted in *S. cerevisiae* by recombinant expression of *easF_Af* from *A. fumigatus* and *dmaW_Aj*, *easE_Aj* and *easC_Aj* from *Aspergillus japonicus* (Fig. 2)*.* The study showed that the peroxisomal targeting signal sequence (PTS-1) in *easC_Aj* was not essential to EasC catalytic activity, while the N-terminal sequence in *easE_Aj* or a secretory signal was needed for EasE activity. Also, overexpression of several native yeast enzymes (pdi1 and ero1: enzymes involved in the formation of disulfide bonds in ER; fad1: a FAD synthase) were shown to improve chanoclavine-I production by 50%, 300%, and 250%, respectively.^77^ In order to extend the EAS pathway in yeast, an engineered *S. cerevisiae* strain, capable of producing chanoclavine-I, was transformed with genes from *A. japonicus* EAS cluster (*easD*, *easA*, and *easG*). This resulted in the production of festuclavine, a downstream intermediate in the EAS pathway. When *easH* from *A. japonicus* was additionally transformed into the yeast strain, shunt product cycloclavine was produced. The proportion of cycloclavine in product mixture was increased with increased *easH_Aj* copy number. This showed that EasH plays an indispensable role in cycloclavine biosynthesis. The maximum biosynthesis of cycloclavine (529 mg L^−1^) was reached in a strain containing multiple copies of genes from the fungal EAS pathway and multiple copies of host genes *pdi1* (protein disulfide isomerase) and *fad1* (FAD synthase). This study showed the potential of *S. cerevisiae* cells as cell factories for the valuable ergot alkaloids.^78^ The recombinant biosynthetic pathway of festuclavine, agroclavine, and cycloclavine from l-tryptophan and DMAPP in *S. cerevisiae* is shown in Fig. 2*.*

### 3.3 Host organism: *Aspergillus nidulans* and *Aspergillus oryzae*

As eukaryotic HE systems, the *Aspergillus* spp. overcome most problems faced by prokaryotic HE systems, circumventing promoter or terminator replacement and intron excision. *Aspergillus nidulans* is a model fungus used in many fields of study. Although the HR frequency in direct gene targeting of *A. nidulans* was low, NHEJ-impaired mutants and heterologous markers were available as early as in 2006, which elevated the HR frequency to 90%.^79^ Thus, *A. nidulans* has been a common host organism for HE of fungal BGCs.

*A. nidulans* was used as the HE system for studying the EAS pathway in *A. fumigatus*. *A. nidulans* naturally does not contain the EAS BGC, nor produces any ergot alkaloids. Chanoclavine-I was produced by the *A. nidulans* mutants transformed with four genes (*dmaW*, *easF*, *easE*, and *easC*) from the EAS cluster of *A. fumigatus* under their native promoters. To investigate the reaction mechanism, additional *A. nidulans* mutants were generated from transformation with incomplete cluster (*dmaW*, *easF*, and *easE*/*easC*). Both strains (WFE and WFC) accumulated N-Me-DMAT instead of chanoclavine-I, indicating that both *easE* and *easC* were necessary for the conversion of N-Me-DMAT into chanoclavine-I. An unidentified novel compound was also observed in the WFC mutant. However, this study does not rule out the possibility that there could be native genes in *A. nidulans* that participated in the biochemical reactions.^80^

Deletion of secondary metabolic gene clusters reduces the biochemical background in the host organism. In an NHEJ-impaired *A. nidulans* strain, the gene clusters for sterigmatocystin, emericellamide, orsellinicacid, asperfuranone, monodictyphenone, and terrequinone were deleted. A *pyrG* marker from *A. fumigatus* was used and recycled repeatedly during the process. Nonreducing polyketide synthase (NR-PKS) gene clusters from *Aspergillus terreus* were cut into smaller fragments, fused with different markers, and transformed into the *A. nidulans* mutant for *in vivo* homologous recombination and the complete NR-PKS sequence was created. The expected product was formed in 17 out of 19 mutants, indicating that most of the DNA design was correct and the gene integration was successful. In order to study the asperfuranone (afo) biosynthetic pathway, the native *afo* gene cluster of *A. nidulans* was deleted. The resulting strains were transformed with various combination of genes from the silent *afo* gene cluster from *A. terreus*. The genes were fused with selected, regulatable promoters. The results showed the order of three genes (*AteafoC*, *D*, and *F*) and their likely roles in the pathway. This study showed that upon deletion of native gene clusters, *A. nidulans* is a candidate host for HE of silent fungal BGCs.^81^

*Aspergillus oryzae* is another popular HE host because it is safe, it is exceptional at producing hydrolytic proteins, and its genome sequence information is available. A wide range of polyketides, nonribosomal peptides, and terpenoids have been synthesized in *A. oryzae* HE systems. Many promoters and selection markers are available; *amyB* has been the most common promoter. A recent review has provided more information about reconstructing fungal biosynthetic gene clusters in the *Aspergillus* spp.^82^

### 3.4 Other expression systems

*Bacillus subtilis* is a Gram-positive bacterium. It is a desirable host for HE because *B. subtilis* is non-pathogenic, its biology is well-understood, and it is known for producing various non-ribosomal peptides (products of the non-ribosomal peptide synthetases, NRPSs) and polyketides. Moreover, the *B. subtilis* strains have been successfully engineered into cell factories for antibiotics.^83–85^ The *eysn* gene encodes for the enniatin synthetase (EYSN) that is responsible for the production of enniatin from d-hydroxyisovaleric acid and amino acids. The *esyn* gene from *Fusarium oxysporum* was placed under an acetoin-inducible promoter system and transformed into *B. subtilis*. Enniatin production reached 1.1 mg L^−1^ in this HE system. This is the first eukaryotic NRPS gene expressed in *B. subtilis*. The yield could potentially be improved by approaches such as metabolic engineering, transcription and translation regulation, and precursor feeding.^86^

*A. fumigatus* is another HE host that has been used for studying the EAS pathway. *A. fumigatus* is a filamentous fungus that innately produces ergot alkaloids. The *easA* gene of *A. fumigatus* was deleted to allow transformation of its homologue (*easA*) and the candidate gene (*easH*/*cloA*) from *Epichloë* sp. Lp1. Transformation of *easA* from *Epichloë* sp. Lp1. resulted in production of agroclavine instead of the wild-type product festuclavine. CloA was found to catalyze multiple steps in the production of lysergic acid from agroclavine.^87^

HE systems of fungal BGCs have also been constructed in *A. oryzae*,^88,89^*A. niger*,^90^ and *N. crassa*.^91^ These organisms can perform eukaryotic post-translational modifications. They will more readily be used as HE hosts once their genomes are decrypted.

A cell-free protein synthesis (CFPS) platform has several advantages such as flexible and controlled expression and reaction environment, no need for host cell engineering, and greater tolerance for toxic wastes. GrsA and GrsB1 are the first two modules of a five-module NRPS protein that synthesizes gramicidin S. GrsA and GrsB1 can catalyze the cyclodimerization to form d-Phe-l-Pro diketopiperazine, a natural shunt product in gramicidin S biosynthetic pathway. GrsA and GrsB1 were expressed in *E. coli*-based CFPS system and purified at ∼106 μg mL^−1^ and ∼77 μg mL^−1^, respectively. d-Phe-l-Pro diketopiperazine was synthesized in a “single-pot” CFPS that co-expressed GrsA and GrsB1 at ∼12 mg mL^−1^.^92^

## 4 Conclusions

The gene manipulation for filamentous fungi is often hindered by inefficient gene targeting. Here we have summarized many available methods for improving the frequency of precise gene targeting. A combination of selective methods has been proven to be very effective in fungi that override traditional methods of gene manipulation. These approaches have allowed the functional characterisation of genes in BGCs and enhanced production of mature products or pathway intermediates^99^ or reduced the generation of by-products.

Heterologous expression has been an option for studying fungal BGCs outside their complex, native hosts. Successful heterologous expression demands apposite choice of host and vector systems, frequently the addition of foreign promoters for better regulation, and the toolkits and technologies helping to streamline the process.^1^ Recent advances in increasing expression levels and product yield often come from gene manipulation in the heterologous host. However, product yield from most reported fungal genetic studies still has great potential to be optimized. The rapidly accumulating genomics information will also continue to benefit fungal genetic research in the future.

Advanced gene targeting and heterologous expression technologies enable fast, convenient, and clear-cut confirmation of gene functions. As more BGCs in the fungal genome are unearthed and elucidated, biosynthesis of valuable fungal products and their derivatives will be achieved, leading to discovery of new candidates of drugs and other biochemical products.

## Conflicts of interest

There are no conflicts to declare.

## Supplementary Material
